# Vaginal metastasis from breast cancer: A case report

**DOI:** 10.1515/biol-2022-0623

**Published:** 2023-06-20

**Authors:** Yunbo Yan, Tianjiao Guo, Minmei Zhang, Guozhong Cui

**Affiliations:** Hebei Medical University, Shijiazhuang, China; Second Department of Thyroid and Breast Surgery, Cangzhou Central Hospital, No. 16 of Xinhua West Road, Yunhe District, Cangzhou, Hebei Province, 061000, China; Department of Diagnostic Computed Tomography, Cangzhou Central Hospital, Cangzhou, China; Vascular Surgery Department, TCM Hospital of Shijiazhuang City, Shijiiazhuang, China

**Keywords:** breast cancer, vaginal metastases, histopathological examination, eribulin, bevacizumab

## Abstract

Breast cancer is one of the most common malignancies in women. However, cases of vaginal metastases of breast cancer are rarely reported in China and abroad. The main clinical symptom of vaginal metastases of breast cancer is vaginal bleeding. This article aims to provide a reference for the diagnosis and clinical management of vaginal metastases from breast cancer. This article describes in detail the management of a 50-year-old woman with vaginal metastases from breast cancer, who was admitted to the hospital with persistent vaginal bleeding without apparent causes. Persistent vaginal bleeding was found after two and a half years when her breast cancer surgery was performed. After comprehensive evaluation, vaginal mass resection was performed. Postoperative histopathology confirmed that the vaginal mass was breast cancer metastasis. The patient was treated with local radiotherapy and three cycles of eribulin and bevacizumab after the vaginal mass was removed. A reexamination of computed tomography showed that the chest wall metastases were less extensive than before. Orbital metastases were also reduced in size, which was revealed by the physical examination. The patient had since failed to return to hospital on time for a regular treatment due to personal reasons. After 9 months of follow-up, the patient died of multiple metastases. The diagnosis of vaginal masses is based on pathological examination, and systemic treatment should be the mainstay when extensive metastases are presented.

## 1 Introduction

Breast cancer is one of the most prevalent malignancies in women worldwide, and it is a major threat to women’s health. Breast cancer can spread to other parts of the body, leading to metastatic breast cancer. According to the statistics of many research reports, 6–60% of breast cancer patients are diagnosed as metastatic breast cancer at an early stage [1]. Metastases to the genital organs are less common, with the ovaries being the common site of metastases to the genital organs, but the vagina is rare [2]. A case of vaginal metastasis from surgically resected, pathologically confirmed breast cancer treated in our hospital is reported as follows.

## 2 Case presentation

A female, aged 50 years, was admitted to the Second Department of Thyroid and Breast Surgery of Cangzhou Central Hospital on July 26, 2019, complaining of a left breast swelling for 1 day. The patient was treated with a modified radical surgery for left breast cancer. According to the postoperative pathological and immunohistochemical results, the patient was diagnosed as triple-negative breast cancer (TNBC). After the surgery, eight cycles of intravenous chemotherapy with AC-T regimen were planned to be performed. Four cycles of intravenous chemotherapy with AC (doxorubicin liposome + cyclophosphamide) regimen and four cycles of intravenous chemotherapy with T (docetaxel) regimen were finally operated. Thereafter, in September 2020, the patient was examined that breast cancer metastasizes to sternum and parasternal soft tissue in a hospital in Beijing. After one cycle of TCb (docetaxel + carboplatin) intravenous chemotherapy, the patient was discharged. On January 29, 2021, during the reexamination at the Second Department of Thyroid and Breast Surgery of Cangzhou Central Hospital, the patient was found to have an increasing range of chest wall recurrence, which was considered to be in progress. It was advised to change the treatment into the chemotherapy regimen to NP (vinorelbine + cisplatin), which was given two cycles. The reexamination on April 16, 2021, showed that tumor was in progress and three cycles of camrelizumab were performed. The reexamination results on July 2021 revealed that there is a possibility of recurrence and chest wall metastasis. The patient was treated with radiotherapy + anlotinib-targeting therapy in a hospital in Tianjin. The patient complained that the chest wall occupancy was smaller than before during the examination in Tianjin in December 2021.

**Figure 1:**
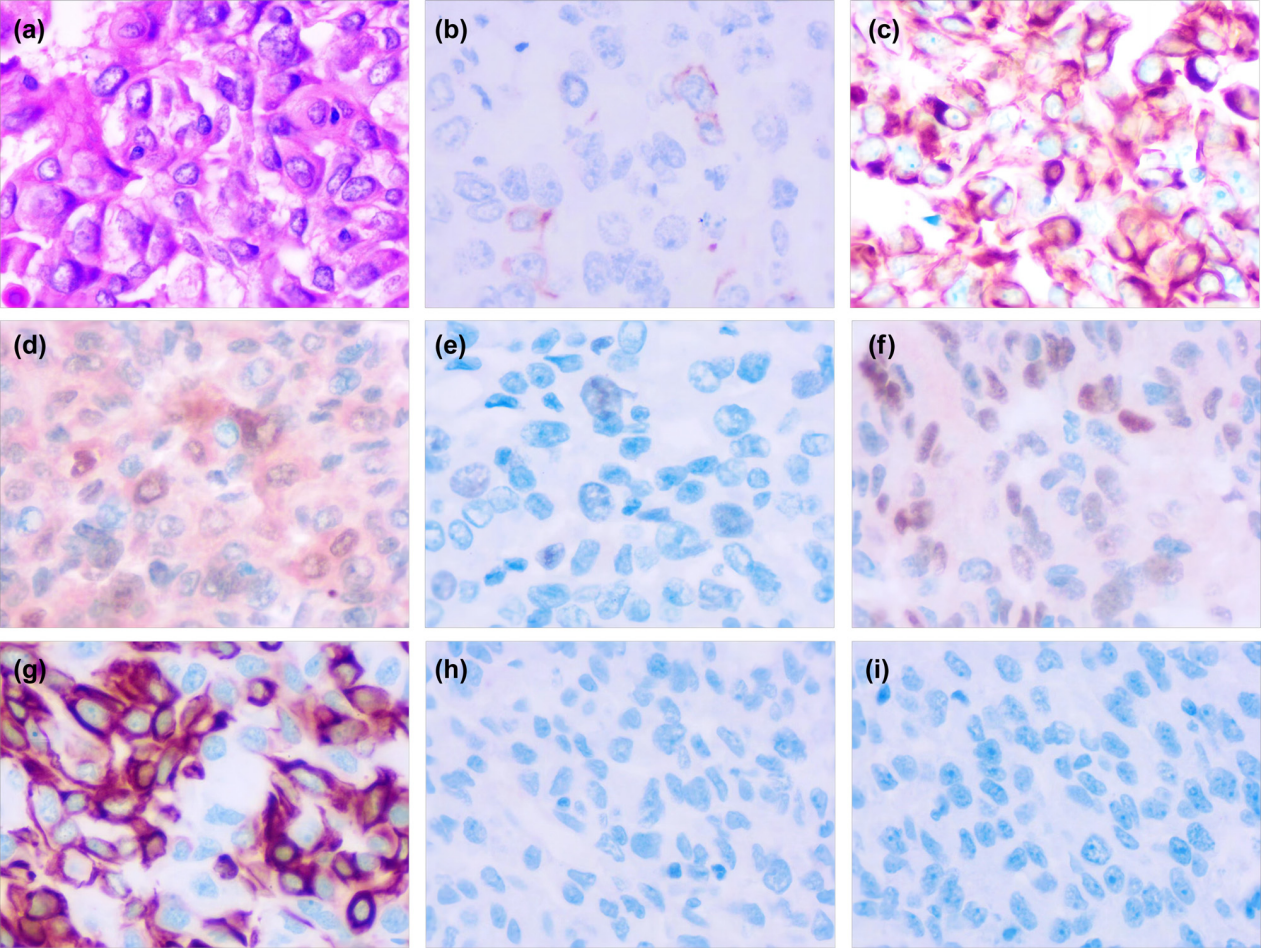
Histopathology of vaginal mass. (a) Hematoxylin and eosin staining (magnification: 400×); (b) immunohistochemistry for CK5/6 (magnification: 400×); (c) immunohistochemistry for CK8/18 (magnification: 400×); (d) immunohistochemistry for P16 (magnification: 400×); (e) immunohistochemistry for Ki-67 (magnification: 400×); (f) immunohistochemistry for GATA3 (magnification: 400×); (g) immunohistochemistry for CK7 (magnification: 400×); (h) immunohistochemistry for ER (magnification: 400×); and (i) immunohistochemistry for PR (magnification: 400×).

The patient was admitted to the First Department of Gynecology of Cangzhou Central Hospital on January 12, 2022, due to abnormal vaginal bleeding for 2 months. Gynecological examination revealed married vulva, vaginal patency, superfluous mass in the upper-third of the right vaginal wall, 3 cm in diameter, 0.8 cm in diameter at the tip, smooth cervix, anterior position of the uterus, normal size, no pressure pain, and no obvious abnormality in the bilateral adnexal area. Pathological results of vaginal mass tissue (Figure 1a): (vaginal wall mass) hypodifferentiated adenocarcinoma with mostly degenerated necrotic cells, not excluding the source of breast in combination with the history and immunohistochemistry, CK5/6 (small focal+) (Figure 1b), CK8/18 (+) (Figure 1c), P16 (partial+) (Figure 1d), P40 (−), Ki-67 (scattered+) (Figure 1e), Vimentin (−), GATA3 (focal+) (Figure 1f), CK7 (+) (Figure 1g), ER <1% (+) (positive control normal) (Figure 1h), PR <1% (+) (positive control normal) (Figure 1i), and Her-2 (0) (positive control normal).

**Figure 2:**
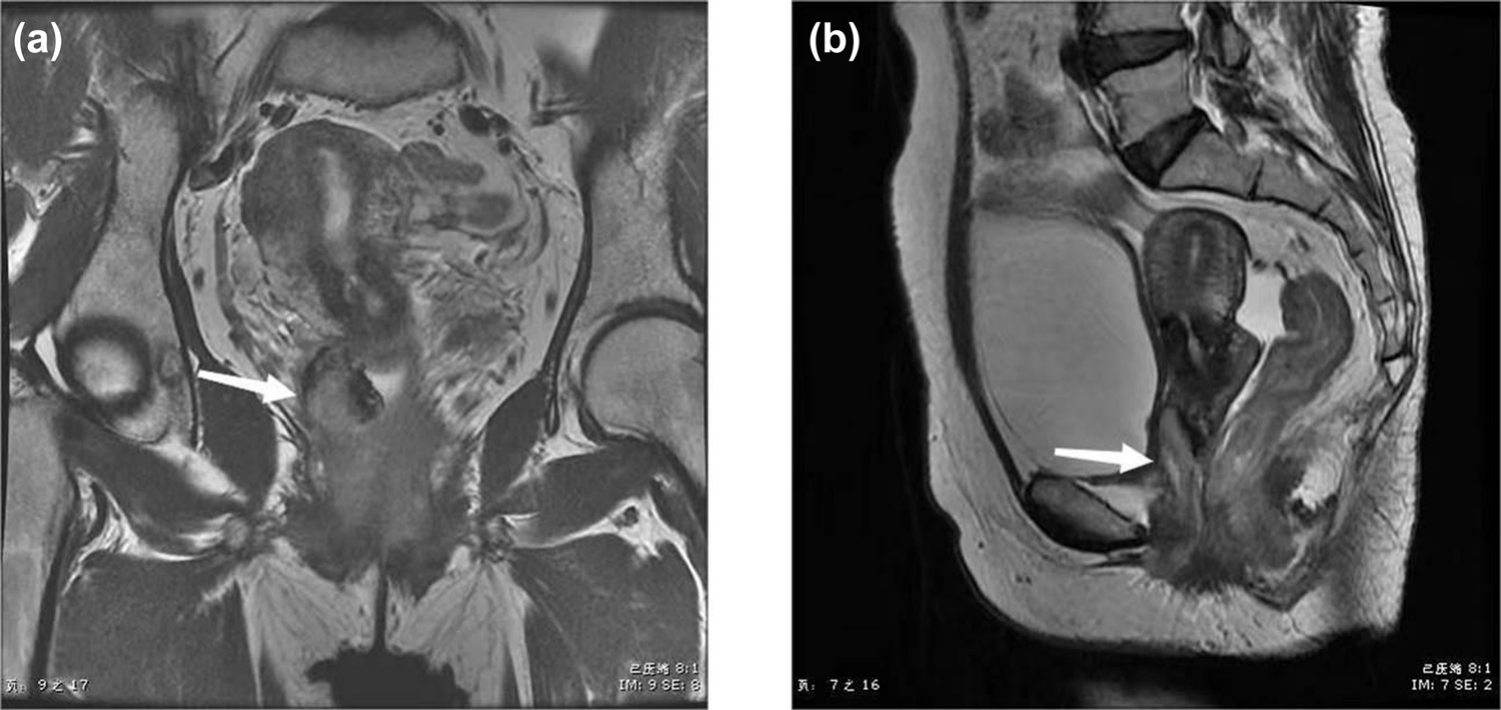
(a and b) T2-weighted coronal and sagittal magnetic resonance imaging (MRI) showed a mass in the right anterior wall of middle and upper segment of vagina (arrowhead).

The pelvic magnetic resonance scan with simultaneous enhancement revealed that (Figure 2): 1. occupancy of the right side of the wall of the upper middle vagina, a polyp was considered, and a biopsy was recommended to exclude malignant changes; and 2. pelvic effusion.

**Figure 3:**
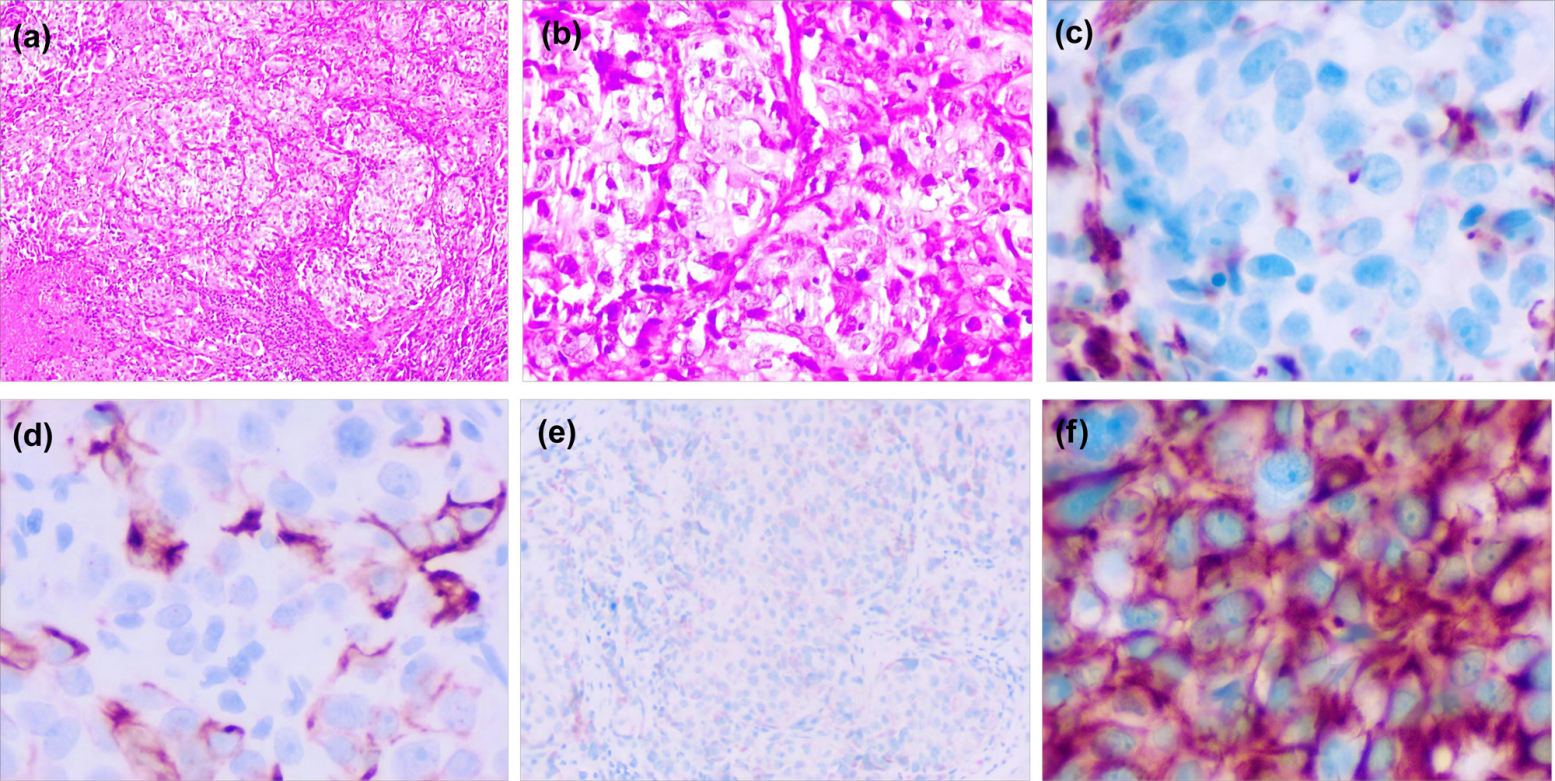
Postoperative histopathology of vaginal wall tumor. (a) Hematoxylin and eosin staining (magnification: 100×); (b) hematoxylin and eosin staining (magnification: 400×); (c) immunohistochemistry for Vimentin (magnification: 400×); (d) immunohistochemistry for CK5/6 (magnification: 400×); (e) immunohistochemistry for Melan-A (magnification: 400×); and (f) immunohistochemistry for CK8/18 (magnification: 400×).

Complete relevant laboratory tests were performed after admission, and there is no obvious surgical contraindication. On January 17, 2020, a vaginal mass was operated under general anesthesia. The mass was mostly detached from the top of the right vaginal fornix, with a residual lesion of about 3 cm in diameter and a residual root of about the size of a copper penny, with a smooth normal cervical area. The tumor was completely removed at the normal tissue 0.5 cm outside the root, and no residual was found at the cutting edge. The patient was found to be recovered well after the operation and was given a blood transfusion to correct the anemia and was discharged 5 days after the operation. Postoperative pathology (Figure 3a and b): (vaginal wall mass) hypofractionated carcinoma, clinical and immunohistochemically considered to be of breast origin, Vimentin (+) (Figure 3c), CK5/6 (+) (Figure 3d), P63 (−), HMB45 (−), Melan-A focal (+) (Figure 3e), S-100 (−), and CK8/18 (+) (Figure 3f).

The patient was transferred to our department after surgery; meanwhile, orbital metastases were also diagnosed. Thereafter, the patient was given orbital radiotherapy followed by three cycles of eribulin and bevacizumab. On May 19, 2022, a CT reexamination showed that there is a reduction in the extent of chest wall metastases compared to the previous one. On examination, the orbital metastases were also smaller than before. The patient has since failed to return to hospital on time for a regular treatment for personal reasons. After 9 months of follow-up, the patient died of multiple metastases.


**Informed consent:** Informed consent has been obtained from all individuals included in this study.
**Ethical approval:** The research related to human use has been complied with all the relevant national regulations, institutional policies, and in accordance with the tenets of the Helsinki Declaration, and has been approved by Ethics Committee of Cangzhou Central Hospital.

## 3 Discussion

The patient’s history, imaging data, postoperative pathology, and immunohistochemical findings led to the diagnosis of postoperative vaginal metastases from breast cancer. The mechanism of breast cancer metastasis to the vagina is still unknown, but considering that the patient had multiple distant metastases to the sternum, parasternal soft tissues, and chest wall, it is more likely that the vaginal metastases came from hematogenous metastases [2]. The common sites of distant metastases from breast cancer are lung, bone, liver, and supraclavicular lymph nodes [2]. Metastases from breast cancer to the genital organs are less common, with the ovaries being the common site of metastases to the genital organs, but the vagina is rare. Primary tumors associated with metastatic vaginal adenocarcinoma are most commonly found in the uterus and rarely in the breast [3]. Primary vaginal cancers account for only 1% of all gynecological malignancies [3]. The majority of primary vaginal cancer cases are squamous cell carcinomas [2,4,5], and other histological types, such as adenocarcinoma, are extremely rare [5]. Therefore, histopathological examination is essential for an accurate diagnosis [4,6]. When a vaginal tumor shows pathologically to be an adenocarcinoma, the possibility of metastasis of the lesion must be considered [5].

Clinical signs of metastasis to the vagina include vaginal bleeding, vaginal masses, vaginal discharge, vaginal staining, and perineal discomfort [3,5]. MRI evaluation is useful in detecting vaginal lesions and distinguishing adenocarcinoma from squamous cell carcinoma. Adenocarcinoma usually has a high T_2_WI signal, whereas squamous cell carcinoma appears as intermediate T_2_WI and low T_1_WI signal on MRI [5].

Vinorelbine, a semi-synthesized vinca alkaloid belonging to the Catharanthus alkaloid group, is a cell cycle-specific agent that exhibits cytotoxicity through binding to tubulin, thereby disrupting the microtubule formation during mitosis [6]. Vinorelbine has no cross-resistance to anthracyclines and taxanes, and it is an effective agent for the treatment of recurrent and metastatic TNBC [6]. Vinorelbine and cisplatin, which act on various targets, exhibit a synergistic anticancer activity and have shown a relatively high efficacy against TNBC [6]. In the 2022 Breast Cancer Diagnosis and Treatment Guidelines issued by Chinese Society of Clinical Oncology, the NP scheme has become a Class 1A recommended combined treatment scheme after the failure of triple-negative taxane treatment for advanced breast cancer [7]. Camrelizumab is a programmed death receptor 1 (PD-1) inhibitor. PD-1 is a member of the CD28 superfamily that is expressed mainly in activated T-lymphocytes and myeloid cells, functioning as a crucial immunosuppressive molecule [8]. The PD-1 mainly consists of an extracellular immunoglobulin variable region, a hydrophobic transmembrane region, and an intracellular region. The tail of the intracellular region has an immunoreceptor tyrosine-based inhibitory motif and an immunoreceptor tyrosine-based switch motif (ITSM). PD-1 is an essential immune checkpoint receptor for activated T cells and plays a critical role in immunosuppression control [8]. Binding to its ligand programmed death ligand-1 (PD-L1) induces the phosphorylation of tyrosine in ITSM of PD-1, which dephosphorylates downstream protein kinases Syk and PI3K, inhibiting the transcription and translation of genes and cellular factors required for T cell activation. Tumor cells can inhibit the killing function of T cells by high expression of PD-L1, thus contributing to immune escape [8]. TNBC is the most immunogenic subtype of breast cancer with higher levels of PD-L1 expression and tumor-infiltrating lymphocytes than other subtypes, suggesting that it is more likely to benefit from treatment with immune checkpoint inhibitors [9]. Anlotinib is a new type of small-molecule antiangiogenic tyrosine kinase inhibitor that targets vascular endothelial growth factor receptor, fibroblast growth factor receptor, platelet-derived growth factor receptors, and c-kit [10]. Anlotinib inhibits cell migration and capillary-like tube formation, and angiogenesis induced by vascular endothelial growth factor (VEGF). Anlotinib also decreased the expression of proangiogenic factors, and enhanced the expression of immune cell adhesion molecules and chemokines and their receptors [10]. It suppressed tumor angiogenesis and normalized the remaining blood vessels [10]. In one study [10], anlotinib was found to show good efficacy and manageable toxicity in patients with metastatic breast cancer who had failed standard treatment. It is emerging as a treatment option for metastatic breast cancer.

Standard therapy for vaginal metastases from breast cancer has not been established. Treatment options for vaginal metastases include surgical resection, radiotherapy, and chemotherapy, both monotherapy and combination therapies [4]. Researchers including Bellati and Filippo have reported the first case of surgical treatment for isolated vaginal metastasis of breast cancer [11]. After 12 months of follow-up after surgery, the patient is still free from disease. It demonstrates that surgery is an effective choice for the treatment of isolated vaginal metastasis of breast cancer. While surgical resection and radiotherapy are commonly used for patients with isolated vaginal metastases [12], systemic therapy is used for patients with extensive metastases [2,4]. Eribulin (Halaven) is a fully synthetic analogue of chondroitin B and is a non- taxane inhibitor of microtubule dynamics. Compared to other microtubule protein-targeting agents such as violet shirts and perillyl alkaloids, eribulin has a unique mode of action. It can inhibit elongation (polymerization) rather than shortening (depolymerization) of microtubules to induce cancer cell death [13]. Bevacizumab is a humanized anti-VEGF monoclonal antibody that controls tumor growth by inhibiting VEGF-A and slowing down the growth of new blood vessels [14]. The efficacy of eribulin and bevacizumab on vaginal metastases could not be assessed as the patient had already had the vaginal metastases removed prior to systemic therapy. Further studies are needed to explore in the treatment of vaginal metastases from breast cancer due to the non-universal nature of this case and the limited evidence to guide treatment.

Vaginal metastases from breast cancer treatment with surgery, chemotherapy, and/or radiation directly affect the reproductive organs and sexual health. Vulvovaginal atrophy (VVA), a direct consequence of estrogen deficiency, is a commonly reported symptom, including symptoms, which are typically progressive and unlikely to resolve spontaneously, such as vulvovaginal dryness, burning or irritation, dyspareunia, or urinary symptoms of urgency, dysuria, or recurrent urinary tract infection [15]. Approximately 50–75% of breast cancer survivors suffer from one or more VVA symptoms [16]. This is even more a problem for patients with vaginal metastasis of breast cancer. Therefore, attention to sexual life and quality of life (QoL) is imperative to the complete care of these women. Overall, in these patients, the available armamentarium encompasses vaginal lubricants, moisturizers, estrogens, dehydroepiandrosterone, CO_2_ laser, ospemifene, and counseling [17]. CO_2_ laser treatment improves blood flow in vaginal tissues, healthy vaginal epithelium, and muscle tone; causes collagen contraction; restores elasticity of the vaginal canal; and improves the extracellular matrix of the mucosal structures and function with findings similar to a premenopausal state [15]. Pieralli et al. in 2016 were among the first to evaluate the use of CO_2_ laser treatment on 50 breast cancer survivors with contraindications to hormonal treatments: the results demonstrated a significant improvement of VVA dyspareunia after three sessions of treatment [18]. Preliminary results of the feasibility EPIONE trial showed that laser therapy reduces vaginal mucosal dryness in women with VVA that developed after systemic breast cancer therapy, but two CO_2_ laser sessions were used [19]. QoL preservation after anticancer therapy is a challenge for vaginal metastases from breast cancer.

An important prognostic factor for patients with vaginal metastases from breast cancer is the presence of secondary lesions in other organs [20], a fact that was observed in this case. When breast cancer presents with vaginal metastases, metastases from other sites have often already occurred; therefore, the prognosis is poor [21] (Table 1).

**Table 1:** Disease development process and the treatment line

Time	Disease development	Treatment
2019.07	Left breast swelling for 1 day	A modified radical surgery for left breast cancer and eight cycles of intravenous chemotherapy with AC-T regimen
2020.09	Breast cancer metastasizes to sternum and parasternal soft tissue	One cycle of TCb (docetaxel + carboplatin) intravenous chemotherapy
2021.01	An increasing range of chest wall recurrence considered as tumor progression	Two cycles of NP (vinorelbine + cisplatin) chemotherapy regimen
2021.04	Tumor progression	Three cycles of camrelizumab
2021.07	Recurrence and chest wall metastasis	Radiotherapy + anlotinib targeting therapy
2022.01	Abnormal vaginal bleeding for 2 months	Excision of vaginal mass
2022.01	Orbital metastases	Orbital radiotherapy followed by three cycles of eribulin and bevacizumab
2022.10	The patient died of multiple metastases	

## 4 Conclusion

Vaginal metastases from breast cancer are very rare. Histopathological examination is essential for an accurate diagnosis. When extensive metastases are present, systemic therapy should be the mainstay. Eribulin in combination with bevacizumab may be effective in the treatment of vaginal metastases from breast cancer.
